# Cementless Hemiarthroplasty for a Femoral Neck Fracture in a 93-Year-Old Female Patient: A Case Report

**DOI:** 10.7759/cureus.68050

**Published:** 2024-08-28

**Authors:** Hatim Mohammed Alshareef, Ahmed Elbarbary, Abdulrahman S Hassan, Faris A Alzahrani

**Affiliations:** 1 Orthopedic Surgery, King Fahad Armed Forces Hospital, Jeddah, SAU; 2 Medicine and Surgery, University of Jeddah, Jeddah, SAU

**Keywords:** cementless hemiarthroplasty, femoral neck fracture, hip fracture management, cemented vs. cementless hemiarthroplasty, case report

## Abstract

Displaced femoral neck fractures are prevalent in the elderly, necessitating surgical intervention to restore function and mobility. Hemiarthroplasty, available in both cemented and cementless forms, is a common treatment. While cemented hemiarthroplasty is traditionally preferred, cementless options are gaining attention for their potential advantages, including reduced operative time, decreased blood loss, and preservation of bone stock for future revisions. This case report details the successful application of cementless hemiarthroplasty in a 93-year-old female with a displaced femoral neck fracture, underscoring its feasibility and potential benefits in the very elderly. The patient, with multiple comorbidities including hypertension, cerebrovascular accident, osteoarthritis, dementia, chronic kidney disease, diabetes mellitus, osteoporosis, and limited mobility, presented with left hip pain following a fall. Radiographs confirmed a displaced femoral neck fracture. Cementless hemiarthroplasty was performed using the lateral Harding approach, achieving stable fixation without cement. Postoperative care involves standard pain management, early mobilization, and monitoring for complications. This case highlights the potential benefits of cementless hemiarthroplasty, such as reduced operative time and decreased risk of cement-related complications, particularly in elderly patients with good bone quality. The successful outcome, characterized by stable fixation and absence of intraoperative or early postoperative complications, emphasizes the importance of individualized patient assessment and tailored surgical approaches. Further research is needed to refine guidelines and expand the evidence base for the use of cementless techniques in this demographic.

## Introduction

Displaced femoral neck fractures are a common injury among the elderly population, often requiring surgical intervention to restore function and mobility [1]. Hemiarthroplasty has emerged as a widely accepted treatment option, with both cemented and cementless techniques being utilized [2]. While cemented hemiarthroplasty has traditionally been the preferred approach, cementless hemiarthroplasty has gained increasing attention due to potential advantages, [3] such as reduced operative time, decreased blood loss, and preservation of bone stock for future revisions [4].

However, the application of cementless hemiarthroplasty in the oldest-old patient population, defined as individuals aged 85 years and above, remains an area of ongoing research and debate. This case report presents the successful use of cementless hemiarthroplasty in a 93-year-old female patient with a displaced femoral neck fracture. The case highlights the feasibility and potential benefits of this technique in the management of hip fractures in the very elderly population.

## Case presentation

The patient is a 93-year-old female with a detailed medical history, including hypertension diagnosed 20 years ago, a cerebrovascular accident 10 years prior, osteoarthritis diagnosed 15 years ago affecting both knees, and age-related hearing impairment. She was diagnosed with Alzheimer's dementia five years ago, chronic kidney disease stage 3, diabetes mellitus type 2 diagnosed 15 years ago, and osteoporosis diagnosed eight years ago. She underwent an open reduction and internal fixation (ORIF) of the right femur three years ago. This surgery was due to a fall (similar to our present case), and recovery was complicated by limited mobility, necessitating the use of a walker. Currently, the patient is on antihypertensives, metformin for diabetes, bisphosphonates for osteoporosis, and donepezil for Alzheimer's.

The patient presented to the ER with left hip and leg pain following a fall at home two days prior. She tripped over a carpet while using her walker. There were no associated symptoms like loss of consciousness or head injury. Her functional status includes limited mobility with a walker, which is maintained with assistance.

On examination, her vital signs were stable. She was alert and oriented. Cardiovascular and respiratory exams revealed normal heart sounds and clear lung fields, respectively. The abdomen was soft and non-tender. Palpable pulses in the lower limbs were present, with no new neurological deficits noted. The neurological assessment was performed due to her dementia and prior stroke history.

A CT brain scan was conducted to rule out any acute changes due to her fall, given her stroke history. It showed no acute hemorrhage or major infarction but noted multiple ischemic insults, a small meningioma, and age-related changes.

**Figure 1:**
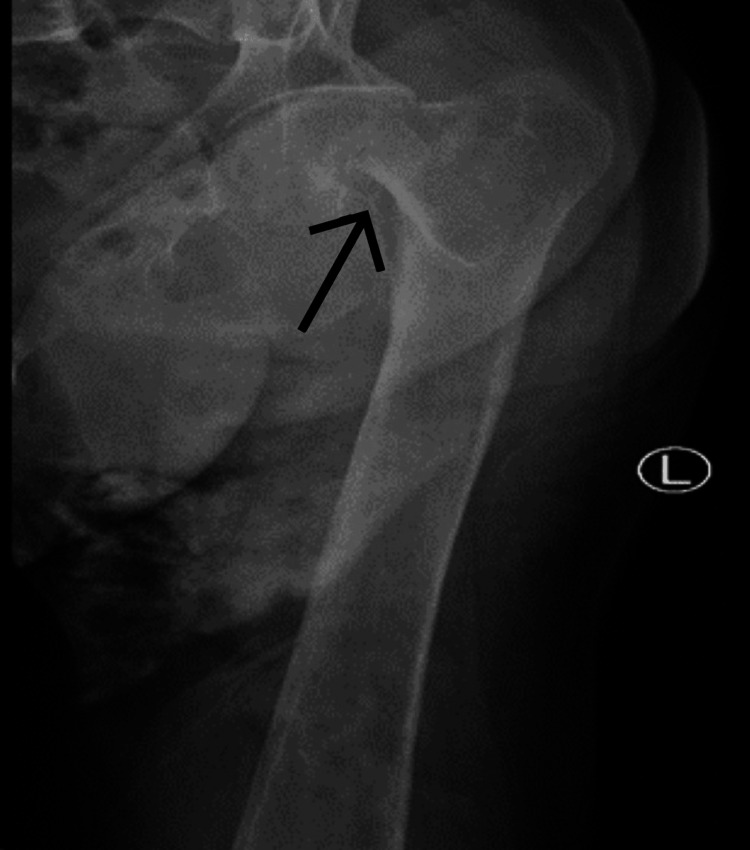
X ray anteroposterior hip shows displace comminuted neck of femur fracture

A pelvic X-ray was performed after the CT scan due to her hip pain, The anteroposterior view of the hip confirms the presence of a displaced femoral neck fracture (Figure 1). The fracture line is visible, and the femoral head is not in its normal anatomical position, indicating the fracture is displaced. 

**Figure 2:**
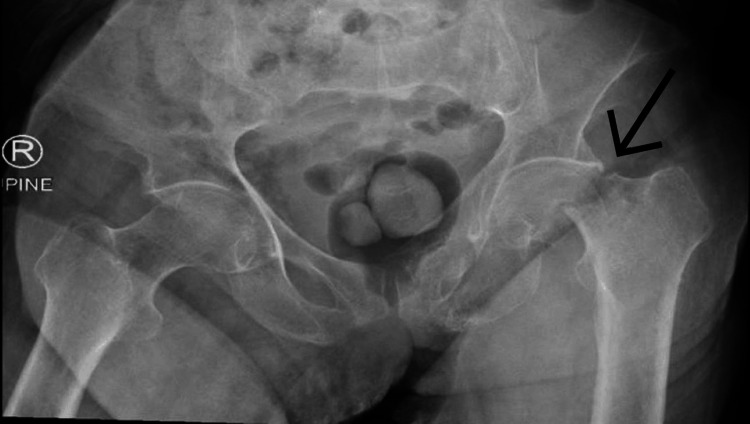
The anteroposterior view of the pelvis shows a clear fracture of the left femoral neck.

X-rays confirmed a left neck of femur fracture (Figure 2). The anteroposterior view of the pelvis shows a clear fracture of the left femoral neck. The fracture appears to be displaced, as indicated by the misalignment of the femoral head with the acetabulum. This type of fracture would require prompt medical attention and likely surgical intervention to properly realign the joint and promote proper healing.

**Figure 3:**
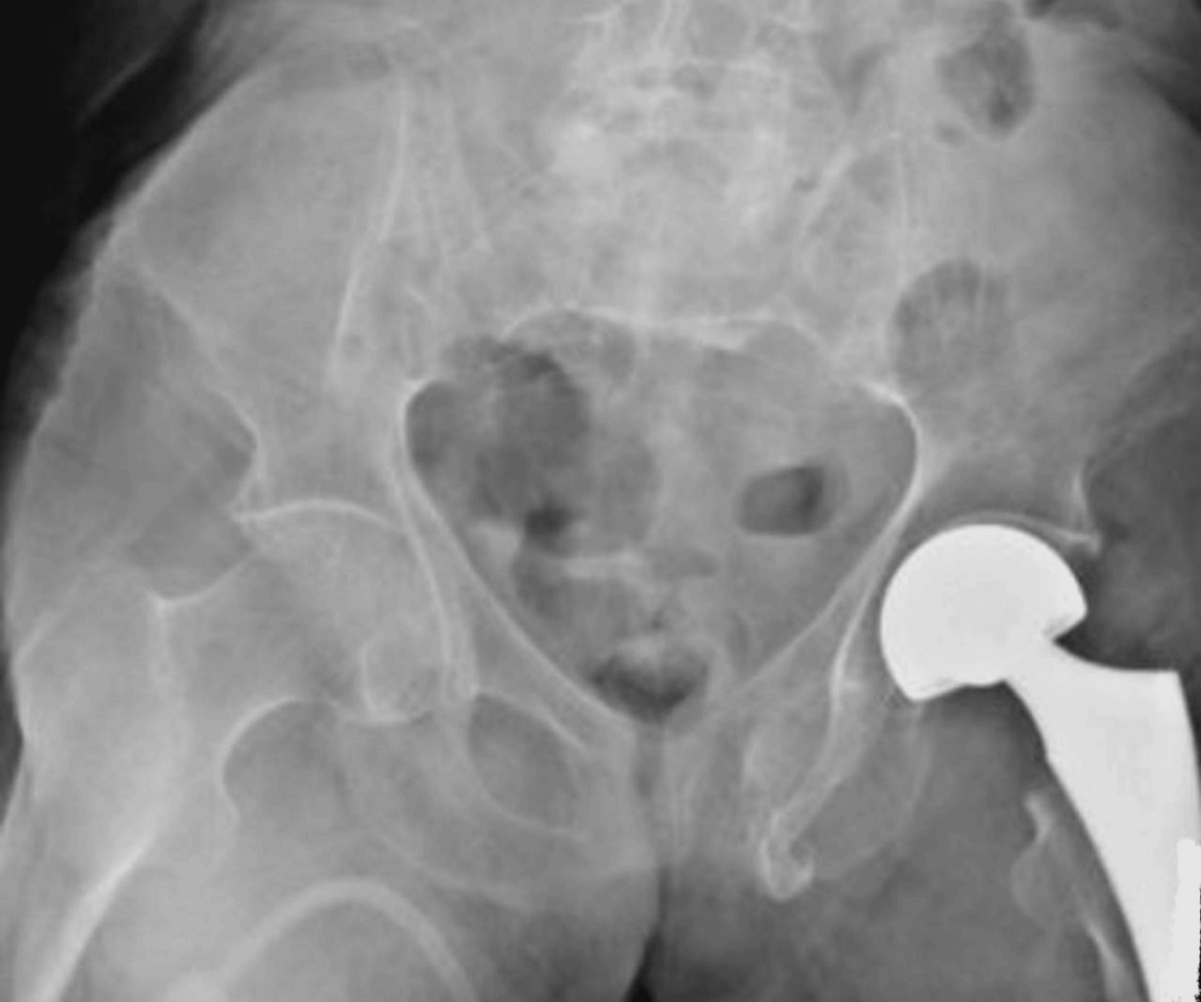
Postoperative X-ray

**Figure 4:**
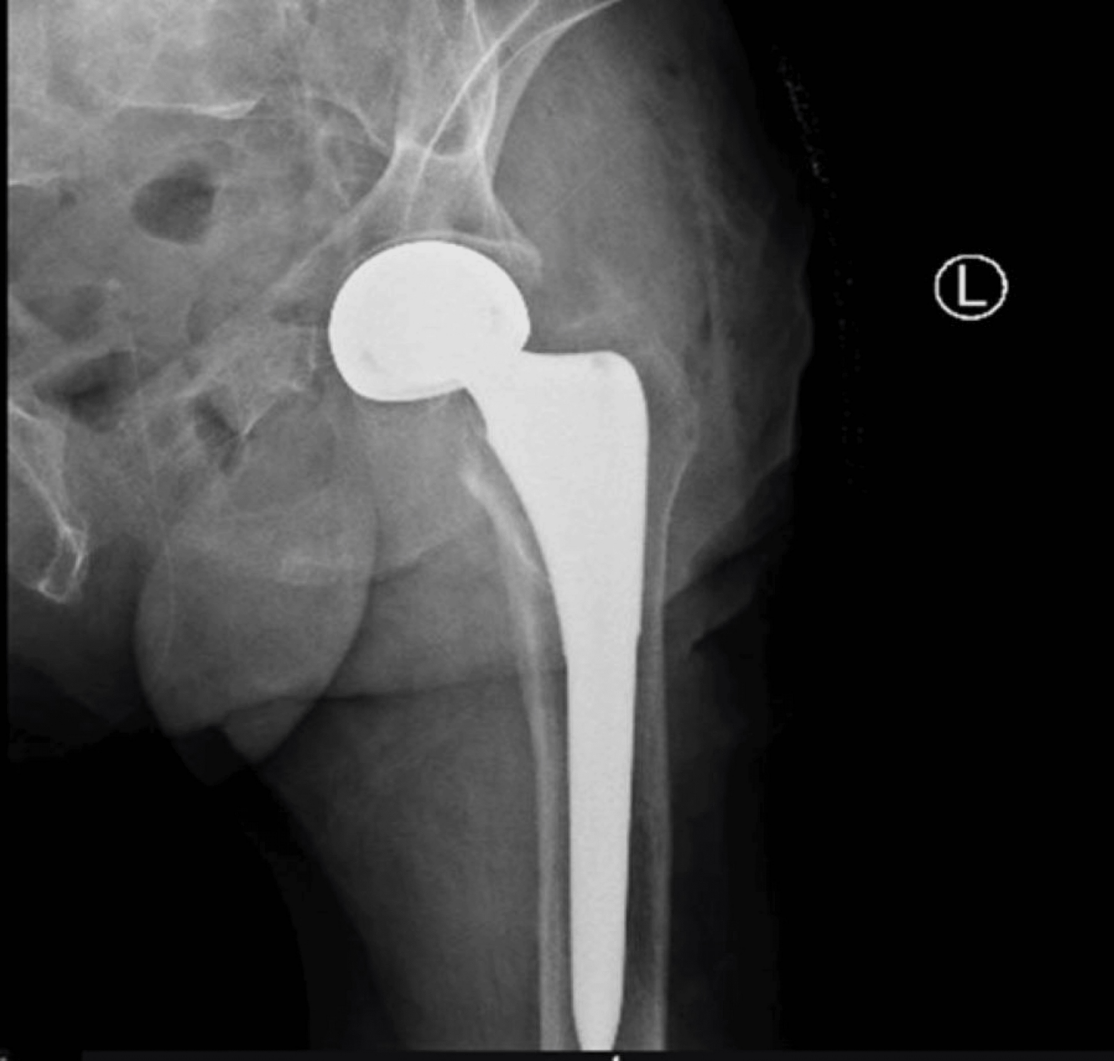
Postoperative anteroposterior X-ray

Cementless hemiarthroplasty was performed (Figures 3-4) to treat the patient's displaced femoral neck fracture. The lateral Harding approach was chosen due to the patient's advanced age (93 years) and comorbidities, as this approach allows for better visualization and access to the femoral neck while minimizing soft tissue dissection. The femoral head was carefully resected, and the acetabulum was inspected to ensure optimal placement of the prosthesis. 

A cementless femoral stem was selected and securely implanted, taking advantage of the patient's good bone quality which permitted reliable initial fixation without the use of cement. This cementless approach was preferred in this elderly patient to avoid the potential complications associated with cement, such as thermal necrosis and impaired remodeling.

Postoperative stability was confirmed through a full range of motion assessment, and meticulous attention was paid to achieve proper leg length and offset to ensure balanced biomechanics. Hemostasis was carefully managed throughout the procedure to minimize blood loss. The wound was closed in layers, starting with the deep tissues, followed by the fascia, subcutaneous tissue, and finally the skin, ensuring a secure and aesthetically acceptable closure.

The patient tolerated the procedure well and was able to begin partial weight-bearing on postoperative Day 2 with physical therapy. At the three-month follow-up, the patient had regained full range of motion and was ambulating independently with the use of a cane. The case highlights the feasibility and potential benefits of cementless hemiarthroplasty in managing hip fractures in the very elderly population, particularly in those with multiple comorbidities.

## Discussion

The management of displaced femoral neck fractures in the elderly, particularly in the oldest-old population, presents unique challenges due to comorbidities and frailty. This case report demonstrates the successful use of cementless hemiarthroplasty in a 93-year-old female patient, highlighting its feasibility and potential benefits in this patient population.

Cementless hemiarthroplasty offers several advantages over the cemented approach, which is particularly relevant for the management of elderly patients with multiple comorbidities. Notably, the cementless technique can reduce operative time and decrease the risk of intraoperative and postoperative complications such as cement-related cardiovascular events and fat embolism syndrome [3,5]. Additionally, cementless techniques preserve bone stock, which is crucial for potential future revisions, an important consideration given the increasing life expectancy even in nonagenarians [4,6]. Studies have shown that cementless hemiarthroplasty can result in better long-term outcomes and reduced rates of prosthesis-related complications compared to cemented techniques [7,8].

The patient's extensive medical history, including hypertension, cerebrovascular accident, osteoarthritis, dementia, chronic kidney disease, diabetes mellitus, osteoporosis, and limited mobility, added significant complexity to the surgical management. Despite these comorbidities, the cementless approach was chosen, guided by her bone quality which allowed for secure initial fixation without cement. This decision aligns with studies suggesting that with appropriate patient selection, cementless hemiarthroplasty can yield excellent outcomes even in the very elderly [4,9]. Furthermore, the absence of cement mitigates the risk of bone cement implantation syndrome, which can be particularly severe in frail elderly patients [10].

The lateral Harding approach provided excellent exposure and minimized soft tissue damage. Postoperative assessments confirmed stability in all ranges of motion, proper leg length, and balanced biomechanics. The meticulous closure in layers and careful management of hemostasis minimized complications, aligning with best practices in surgical techniques to enhance recovery [1,11].

While cemented hemiarthroplasty remains a widely used technique, particularly favored for its immediate stability and ability to accommodate poorer bone quality [2], the present case demonstrates that in selected patients with adequate bone quality, cementless hemiarthroplasty can provide comparable, if not superior, outcomes. The debate between cemented versus cementless approaches continues, with ongoing research needed to refine patient selection criteria and optimize surgical outcomes [3,12]. The patient's positive outcome, with no intraoperative or early postoperative complications, highlights the importance of a tailored approach considering individual patient factors.

Early mobilization and comprehensive postoperative care, including pain management and physical therapy, were crucial in facilitating recovery and functional improvement. This approach aligns with enhanced recovery after surgery (ERAS) protocols, which have been shown to improve outcomes in elderly patients undergoing hip surgery [13]. Recent meta-analyses have indicated that cementless hemiarthroplasty may be associated with lower rates of perioperative complications and better functional outcomes in selected elderly patients [14,15].

Long-term outcomes following hemiarthroplasty in the elderly are a critical consideration, particularly given the increasing life expectancy and the potential for subsequent revisions. Studies have shown that cementless hemiarthroplasty can result in durable fixation and satisfactory functional outcomes over the long term [16]. The preservation of bone stock with cementless techniques may also facilitate future revision surgeries, which can be more complex in older patients [17]. Additionally, the use of modern implant designs and materials has further improved the longevity and performance of cementless prostheses [18].

The success of cementless hemiarthroplasty is not solely dependent on the surgical technique but also on comprehensive postoperative care and rehabilitation. Early mobilization is a key component in enhancing recovery and minimizing complications such as deep vein thrombosis and pulmonary embolism.

## Conclusions

This case report demonstrates the successful use of cementless hemiarthroplasty in the management of a hip fracture in a very elderly patient with significant comorbidities. The patient's positive functional outcome and rapid recovery highlight the potential benefits of this approach, even in high-risk individuals. The case provides further evidence supporting the feasibility and consideration of cementless techniques for hip fracture treatment in the very elderly population when appropriate patient selection and individualized assessment are employed.

Additionally, this case report conforms with the existing literature on the feasibility and potential benefits of cementless hemiarthroplasty in the elderly, while also providing a new example of positive outcomes in a patient with considerable comorbidities. This expands the evidence base beyond what has been previously reported. Further studies are warranted to continue exploring the use of cementless techniques in this demographic and to help refine guidelines for their appropriate application.
